# Maintenance of Dose-Dependent Bilirubin Reduction with Ultra-High-Irradiance Phototherapy up to 148 μW/cm^2^/nm in Severe Neonatal Jaundice

**DOI:** 10.3390/children13101318

**Published:** 2026-09-27

**Authors:** Yo Shimizu, Shin Kato, Kazuyuki Yamamoto, Masami Asai, Shinji Saitoh, Osuke Iwata

**Affiliations:** 1 Department of Pediatrics and Neonatology, Nagoya City University Graduate School of Medical Sciences, Nagoya 467-8601, Japan; y-shimizu@daidohp.or.jp (Y.S.); ykaz79@med.nagoya-cu.ac.jp (K.Y.); ss11@med.nagoya-cu.ac.jp (S.S.); 2 Department of Pediatrics, Daido Hospital, Nagoya 457-8511, Japan; m-asai@daidohp.or.jp; 3 Department of Pediatrics, Japanese Red Cross, Aichi Medical Center, Nagoya Daini Hospital, Nagoya 466-8650, Japan; skato2@nagoya2.jrc.or.jp

**Keywords:** bilirubin, exchange transfusion, jaundice, phototherapy, irradiance, bilirubin reduction

## Abstract

**Highlights:**

**What are the main findings?**

- Phototherapy irradiance up to 148 μW/cm^2^/nm was associated with a dose-dependent increase in bilirubin reduction in neonates with severe jaundice.
- Ultra-high-irradiance phototherapy was associated with limited short-term adverse events observed in this cohort.

**What are the implications of the main findings?**

- Higher irradiance phototherapy may enhance the effectiveness of phototherapy in neonates with severe jaundice.
- These findings highlight the importance of optimizing phototherapy irradiance in the treatment of severe neonatal jaundice.

**Abstract:**

Background/Objectives: Phototherapy is an effective first-line treatment for neonatal jaundice. Previous studies have demonstrated a linear dose-dependent relationship between irradiance and bilirubin reduction during intensive phototherapy up to approximately 55 μW/cm^2^/nm, although others have suggested that the therapeutic effect plateaus with higher irradiance. However, the efficacy and safety of treatment at these irradiance levels remain unclear. By focusing on newborns with severe jaundice close to the exchange transfusion threshold, we aimed to examine whether the dose-dependent association between phototherapy irradiance and bilirubin reduction is maintained at irradiance levels above 55 μW/cm^2^/nm. Methods: Here, phototherapy delivered at estimated irradiance levels >55 μW/cm^2^/nm was defined as ultra-high-irradiance phototherapy. We retrospectively reviewed 92 neonates with severe jaundice admitted to a tertiary neonatal intensive care unit between 2017 and 2024. Severe jaundice was defined as total serum bilirubin levels above or within 2.5 mg/dL of the exchange transfusion threshold. We evaluated the dependence of the bilirubin reduction rate on estimated irradiance and other clinical variables. Results: Only one neonate required exchange transfusion. In multivariable analysis, the bilirubin reduction rate was associated with estimated irradiance (*p* < 0.001) and total serum bilirubin at phototherapy initiation (*p* < 0.001). Bilirubin reduction was dose-dependent on estimated irradiance up to 148 μW/cm^2^/nm (0.31 mg/dL/h per 100 μW/cm^2^/nm). Transient hyperthermia occurred in 12% of infants. Conclusions: Higher estimated irradiance was associated with greater bilirubin reduction up to 148 μW/cm^2^/nm. These findings support further prospective evaluation of optimized phototherapy irradiance in severe neonatal jaundice.

## 1. Introduction

Neonatal jaundice is one of the most common conditions in the neonatal period, affecting at least 60% and 80% of full-term and preterm neonates, respectively [1]. Severe jaundice requiring treatment is more frequent in infants of East Asian parents than in those of other races [2]. Established regimens using phototherapy and exchange transfusion aim to prevent bilirubin encephalopathy and subsequent kernicterus [3]. Nonetheless, kernicterus still occurs in approximately 0.4 per 100,000 newborns in Japan [4], highlighting the importance of early detection of severe jaundice and timely initiation of effective therapy. Phototherapy is the current first-line treatment and provides rapid bilirubin reduction in most cases [3]. However, approximately 0.5 neonates per 10,000 live births are refractory to standard phototherapy and subsequently require exchange transfusion to directly remove circulating bilirubin [5]. Given the high cost, requirement for a substantial workforce, and risk of adverse events associated with transfusion [6], preventing bilirubin encephalopathy with phototherapy alone would be ideal.

According to the American Academy of Pediatrics, intensive phototherapy requires irradiance of at least 30 μW/cm^2^/nm, whereas conventional phototherapy provides 8–10 μW/cm^2^/nm [3]. Previous studies have reported the use of double-directional phototherapy to enhance the therapeutic effect in neonates with severe jaundice [7,8]. Vandborg et al. observed linear relationships between phototherapy irradiance and the bilirubin reduction rate up to 20–55 μW/cm^2^/nm [9]. Thus, 55 μW/cm^2^/nm represents the upper irradiance level up to which a linear dose–response relationship has previously been demonstrated, whereas the efficacy of “ultra-high-irradiance phototherapy” with irradiance greater than 55 μW/cm^2^/nm remains unclear. Recent phototherapy devices can provide even higher irradiance levels exceeding 55 μW/cm^2^/nm; however, it remains unclear whether the dose-dependent therapeutic effect is maintained at these levels, especially because Tan suggested the presence of a saturation point beyond which high irradiance no longer improves the efficacy of phototherapy [10]. In addition, adverse events associated with high-irradiance phototherapy need to be clarified, given that even standard phototherapy is associated with adverse events, such as hyperthermia, dehydration, and hypocalcemia [11].

By focusing on newborns with severe jaundice close to the exchange transfusion threshold, this study aimed to examine whether the dose-dependent association between phototherapy irradiance and bilirubin reduction is maintained at irradiance levels above 55 μW/cm^2^/nm. We hypothesized that the bilirubin reduction rate would continue to increase with increasing irradiance above this level.

## 2. Materials and Methods

### 2.1. Study Population

This single-center, retrospective observational study was conducted from January 2017 to December 2024. We reviewed data from 620 newborns who received phototherapy in the neonatal intensive care unit (NICU) at Nagoya City University Hospital. Of these, 92 infants with severe jaundice, defined as total serum bilirubin above or within 2.5 mg/dL of the domestic exchange transfusion threshold based on the Nakamura criteria, were included (Figure 1). The 2.5 mg/dL margin was selected to identify infants clinically close to the exchange transfusion threshold, considering that bilirubin may rise rapidly at a rate of 0.5 mg/dL/h or greater in severe hyperbilirubinemia and that, in Japan, preparation for exchange transfusion typically requires four to five hours. In our hospital, transcutaneous bilirubin is measured (Jaundice Meter JM-103, Konica Minolta, Tokyo, Japan) in all newborns every 24 h after birth, regardless of NICU admission. When the transcutaneous bilirubin value exceeds the phototherapy threshold, serum bilirubin is measured to determine treatment. Total serum bilirubin levels were used in all infants to determine the indication for phototherapy and to assess the response after phototherapy initiation. Indications for phototherapy and exchange transfusion followed thresholds proposed by Imura and Nakamura et al., respectively (Table 1) [12,13]. Blood sampling was then repeated every 24 h.

### 2.2. Data Collection

For infants in the final cohort, total serum bilirubin was recorded approximately 24 h before (T_−24_), at (T_0_), and 24 h after (T_+24_) the start of phototherapy. In four infants (4%) without a total serum bilirubin measurement at T_−24_ before NICU admission, the measured transcutaneous bilirubin value at T_−24_ was used as a substitute to calculate the pre-treatment bilirubin rise rate, based on the reported correlation between transcutaneous bilirubin and total serum bilirubin before phototherapy [14]. Transcutaneous bilirubin was not used to assess the response after phototherapy initiation. We extracted gestational age, birth weight, sex, mode of delivery, Apgar scores, umbilical cord blood pH, breastfeeding status, body weight, hemolytic disease due to blood group incompatibility, total serum bilirubin at T_−24_/T_0_/T_+24_, phototherapy mode, and subsequent exchange transfusion from the electronic medical records. Blood group incompatibility and associated hemolytic disease were evaluated based on maternal and neonatal ABO and Rh blood types, direct and indirect antiglobulin tests, and, when clinically indicated, antibody elution testing, together with the clinical course and laboratory findings suggestive of hemolysis. We then calculated the difference between total serum bilirubin and the exchange transfusion threshold, the exact intervals between T_−24_, T_0_, and T_+24_, and body weight loss from birth to T_0_. Potential phototherapy-related adverse events were collected, including hyperthermia (>38 °C), prolonged oliguria (<1 mL/kg/h for >24 h), and excessive weight loss (>10% of birth weight) by T_+24_. Depending on clinical status and feeding tolerance, infusion therapy was administered at the clinician’s discretion. For infants followed up in the outpatient clinic after discharge, available information on potential long-term adverse events was recorded, including skin lesions, cerebral palsy, hematologic malignancy, and solid tumors. Follow-up duration varied among infants according to clinical need and was not standardized for long-term adverse-event surveillance. Adverse events not documented in the medical records were not systematically assessed.

### 2.3. Phototherapy Device

We used LED-based phototherapy devices: NeoBlue (Natus Medical Inc., Middleton, WI, USA; wavelength 450–470 nm) or Bililux (Dräger Japan, Tokyo, Japan; wavelength 460–490 nm). NeoBlue was the primary device from January 2017 to December 2022; Bililux was introduced in January 2022. NeoBlue provides low- and high-irradiance modes; in this cohort, only the high mode was used, with single- or double-direction illumination. Bililux allows linear adjustment of light intensity across five levels (1/5 to 5/5); level 3/5 (60%) or 5/5 (100%) was predominantly used, with single or double direction. The phototherapy configuration was chosen according to jaundice severity: single direction for cases below the exchange transfusion threshold and double direction for those above. During phototherapy, infants were placed supine or prone, wore only a diaper without clothing, and received eye protection. Phototherapy was applied vertically from a single direction or, for double-direction phototherapy, using two devices positioned at approximately 30° on either side of the vertical axis, with a device-to-infant distance of 30 cm in all cases. Incubator use was determined by clinical condition. Irradiance was not measured directly at the infant skin surface. During phototherapy, body temperature, urine output, hydration/body weight, and the prescribed device-to-infant distance were routinely monitored, and incubator temperature was adjusted as clinically indicated. The effective irradiance (*I_eff*) was estimated from manufacturer-reported values and the irradiation angle using the formula *I_eff* = *I*_0_ × *cos θ*, where *I*_0_ is the irradiance at vertical incidence, and *θ* is the angle of incidence following Lambert’s cosine law. Estimated irradiance for NeoBlue (high mode) was 35.5 μW/cm^2^/nm (single) and 60.6 μW/cm^2^/nm (double). For Bililux, estimated irradiance was 51.3 μW/cm^2^/nm at level 3/5 (single), 85.5 μW/cm^2^/nm at level 5/5 (single), and 148 μW/cm^2^/nm at level 5/5 (double) [15,16]. For the purposes of this study, phototherapy delivered at estimated irradiance levels >55 μW/cm^2^/nm was classified as ultra-high-irradiance phototherapy, whereas treatment at ≥30 μW/cm^2^/nm followed the conventional definition of intensive phototherapy.

### 2.4. Data Analysis

The distribution of continuous variables was assessed before analysis. Values are presented as the mean (standard deviation) for approximately normally distributed variables, median (interquartile range) for skewed variables, or number (%), as appropriate. For umbilical cord blood pH and Apgar score, 2% of values were missing; no other variables had missing data. Multiple imputation was performed using fully conditional specification with five imputed datasets. Given the very small amount of missing data, five imputations were considered sufficient for the present analysis. The bilirubin increase rate was calculated as (T_0_–T_−24_) divided by the actual time interval (hours), and the reduction rate as (T_0_–T_+24_) divided by the actual time interval (hours). Univariable regression assessed crude associations between the reduction rate and clinical variables. Multivariable regression evaluated independent predictors of the reduction rate; birth weight (per 100 g), total serum bilirubin at T_0_, time point of total serum bilirubin measurement (hours), phototherapy type and dose, and estimated irradiance were entered a priori. Residual distributions were examined, and multicollinearity was assessed using variance inflation factors. An exploratory analysis compared the reduction rate across five device/power/direction combinations using Welch’s one-way ANOVA, followed by the Games–Howell post hoc test.

All analyses were performed in SPSS Statistics, version 29 (IBM Corp., Armonk, NY, USA).

### 2.5. Ethics Approval

This study protocol was reviewed and approved by the Ethics Committee of Nagoya City University Graduate School of Medical Sciences (approval number: 60-25-0205). The requirement for written informed consent was waived by the Ethics Committee of Nagoya City University Graduate School of Medical Sciences because of the retrospective nature of the study and the use of anonymized clinical data.

## 3. Results

Among 2519 infants admitted to the NICU between January 2017 and December 2024, 620 received phototherapy for jaundice. After excluding two infants with major congenital abnormalities and one who died during hospitalization, 92 infants who met the criteria for severe jaundice were included in the final analysis (Figure 1).

### 3.1. Patients’ Background

The mean gestational age and birth weight were 37.7 (2.5) weeks and 2704 (620) g, respectively. Overall, 17% of neonates were delivered by cesarean section. Body weight change from birth to T_0_ was −3.7% (4.8). At T_0_, 59% received breast milk, and 66% received intravenous infusion. The prevalence of hemolytic disease was 8%. NeoBlue was used in 41 infants: 32 received high-mode single-direction and 9 received high-mode double-direction phototherapy. Bililux was used in 51 infants: 3 received level 3 single-direction, 28 received level 5 single-direction, and 20 received level 5 double-direction phototherapy. Serum bilirubin was not assessed at T_−24_ in four infants (4%); in these cases only, the measured transcutaneous bilirubin value at T_−24_ was used as a substitute. Total serum bilirubin levels were 11.7 (3.9) mg/dL at 51.9 (36.6) h (T_−24_), 17.7 (3.9) mg/dL at 74.6 (47.2) h (T_0_), and 12.3 (2.6) mg/dL at 95.1 (47.6) h after birth (T_+24_). The difference between total serum bilirubin at T_0_ and the exchange transfusion threshold was −0.72 (2.0) mg/dL. The bilirubin increase rate from T_−24_ to T_0_ was 0.36 (0.21) mg/dL/h. The bilirubin reduction rate from T_0_ to T_+24_ was 0.24 (0.23) mg/dL/h. Exchange transfusion treatment was required in one neonate (1%), whose total serum bilirubin increased from 15 mg/dL to 22 mg/dL within 12 h during double-direction phototherapy using the Bililux device.

Hyperthermia occurred in 11 infants during phototherapy and resolved promptly in all cases after adjusting incubator settings and insulation. Excessive body weight loss (>10% of birth weight) by T_+24_ occurred in four infants; however, the mean body weight loss during phototherapy was −3%. Prolonged oliguria (<1 mL/kg/h for >24 h) was not observed. All infants underwent automated auditory brainstem response screening before discharge; unilateral failure occurred in only one infant. Outpatient follow-up was available for 74 of 92 neonates, including 41 followed for >12 months. Among those followed, no blood cancer, solid tumor, skin lesion, or neurological finding suggesting kernicterus was recorded during the available follow-up period (Table 2).

### 3.2. Dependence of Bilirubin Reduction Rate on Clinical Variables

In the univariable analysis, the bilirubin reduction rate was positively associated with birth weight (B, 0.008; 95% confidence interval [CI], 0.001–0.015, per 100 g; *p* = 0.050), total serum bilirubin at T_0_ (B, 0.031; 95% CI, 0.024–0.037; *p* < 0.001), age at T_0_ (B, 0.002; 95% CI, 0.001–0.002; *p* < 0.001), difference between total serum bilirubin and the exchange transfusion threshold (B, 0.019; 95% CI, 0.002–0.036; *p* = 0.03), phototherapy device (Bililux vs. NeoBlue) (B, 0.135; 95% CI, 0.069–0.200; *p* < 0.001), and estimated irradiance level (B, 0.243; 95% CI, 0.141–0.345, per 100 μW/cm^2^/nm; *p* < 0.001) (Table 3). The comparison across the five device/power/direction groups shown in Figure 2 was exploratory; some groups contained very few infants, and the group-specific estimates should therefore be interpreted cautiously (Figure 2).

Multivariable analysis showed that the bilirubin reduction rate was associated with birth weight (B, −0.006; 95% CI, −0.012 to 0.000, per 100 g; *p* = 0.040), total serum bilirubin at T_0_ (B, 0.040; 95% CI, 0.029–0.051; *p* < 0.001), and the time point of total serum bilirubin measurement at T_0_ (B, 0.001; 95% CI, 0.00–0.002; *p* = 0.034) (Table 4).

To test whether the phototherapy device remained associated with treatment efficacy after accounting for irradiance, we developed an alternative multivariable model that included the device as a fixed variable. In this model, the estimated irradiance level (B, 0.220; 95% CI, 0.117–0.324, per 100 μW/cm^2^/nm; *p* < 0.001), but not device (B, −0.017; 95% CI, −0.106 to 0.071, Bililux vs. NeoBlue; *p* = 0.699), was associated with the bilirubin reduction rate (Table 4). Residual diagnostics showed no apparent major deviations, and all VIFs were <5, indicating no substantial multicollinearity.

## 4. Discussion

In this single-center, retrospective observational study of severe neonatal jaundice near the exchange transfusion threshold, we found that the bilirubin reduction rate increased dose-dependently with ultra-high-irradiance phototherapy up to 148 μW/cm^2^/nm. Considering that higher total serum bilirubin at the start of phototherapy was associated with greater bilirubin reduction, our findings suggest that severe jaundice is linked to a good phototherapy response while also conferring increased risks of exchange transfusion and kernicterus. Transient hyperthermia was the most frequent short-term adverse event observed. Because only one infant underwent exchange transfusion and long-term adverse events were not systematically assessed, the effects on the need for exchange transfusion and on long-term safety should be elucidated in future studies.

Tan et al. suggested the presence of a saturation point beyond which further increases in phototherapy irradiance no longer improve the efficacy of bilirubin reduction [10]. Vandborg et al. reported that, in neonates with gestational age >33 weeks, high-irradiance LED phototherapy (20–55 μW/cm^2^/nm) produced a linear increase in bilirubin reduction with increasing irradiance, without a saturation point within that range [9]. Several studies have shown that dual-direction phototherapy from above and below using multiple devices (8–104.3 μW/cm^2^/nm) increases irradiance and exposed body surface area compared with single-direction phototherapy, thereby enhancing total serum bilirubin reduction [7,8]. Whereas prior studies examined irradiance up to 55 μW/cm^2^/nm, our data extended beyond this range and showed that higher estimated irradiance remained associated with a greater bilirubin reduction rate. This association, rather than differences among individual treatment groups, represents the primary finding of the present study.

The bilirubin reduction rate is theoretically determined by the balance among bilirubin production, catabolism, clearance, and phototherapy efficacy. Physiological and pathological conditions such as glucose-6-phosphate dehydrogenase deficiency, ABO hemolytic disease, and hyperemia increase bilirubin production [17,18]. Delayed bilirubin catabolism owing to breast milk components [19] and increased enterohepatic circulation from reduced intake and delayed stooling may diminish phototherapy efficacy. As previously discussed, phototherapy efficacy predominantly depends on irradiance level and exposed body surface area [3]. In our study, greater birth weight was associated with a smaller bilirubin reduction rate during phototherapy, consistent with the findings of Vandborg et al. [9]. The association with birth weight may reflect differences in body surface area-to-weight ratio, circulating blood volume, maturity, skin thickness, and hemoglobin mass, rather than birth weight itself. However, exposed body surface area was not directly measured in this study, and therefore, these mechanisms remain speculative. The change in the direction of the birth-weight coefficient after multivariable adjustment may also partly reflect its correlation with total serum bilirubin levels at phototherapy initiation [9].

We also found that higher bilirubin at the commencement of phototherapy was positively associated with a greater bilirubin reduction rate, consistent with observations from the 1980s [10]. At higher total bilirubin levels, saturation of albumin-binding sites increases the amount of free bilirubin that diffuses into dermal capillaries [20,21] and is rapidly photoisomerized during phototherapy, potentially altering the photochemical conversion of bilirubin [22].

In hyperbilirubinemia secondary to hemolytic disease, the bilirubin reduction rate during phototherapy is reduced compared with non-hemolytic disease owing to excessive bilirubin production from red blood cell destruction [23]. However, the number of infants with hemolytic disease was limited, and the cause of hyperbilirubinemia could not be fully classified in all infants. Therefore, reliable etiology-stratified analyses were not possible. In addition, feeding volume, hydration status, albumin concentration, and severity of hemolysis were not consistently available and may have contributed to residual confounding. Similarly, we did not observe an influence of breast milk feeding on the bilirubin reduction rate, unlike previous reports of a negative impact on phototherapy efficacy [24]. Breast milk inhibits intestinal UGT1A1 activity, maintaining higher unconjugated bilirubin [25]. However, given that the amount of breast milk remained at 26.4 mL/kg/day (range, 0–105) at the start of phototherapy, the age range studied may have been too early to detect a noticeable impact of breastfeeding on phototherapy efficacy.

Phototherapy has short-term complications such as hyperthermia, dehydration, and hemoconcentration; most are mild and require no intervention [26]. Wickremasinghe et al. reported a possible association between neonatal phototherapy and increased long-term cancer risk; however, the signal disappeared after excluding children with Down syndrome, suggesting that Down syndrome may be an upstream condition that increases both the need for phototherapy and the incidence of cancer [27]. In principle, visible light has minimal direct interaction with DNA in dermal cells, and its carcinogenic potential is negligible compared with ultraviolet radiation. Moreover, blue LED phototherapy devices do not emit wavelengths associated with DNA damage [28]. Nonetheless, the present study did not systematically assess ocular effects, DNA-related effects, or other uncommon or long-term adverse events. Given the limited sample size and incomplete, nonuniform follow-up, no firm conclusion regarding long-term safety can be drawn, and continued surveillance is warranted.

Previous studies have shown that intensive phototherapy alone is as effective as exchange transfusion in preventing bilirubin encephalopathy while also shortening hospitalization and reducing costs [29,30]. In Myanmar, intensive phototherapy reportedly prevented exchange transfusion in 33–60% of infants with severe neonatal hyperbilirubinemia [31]. Only one infant in the present cohort underwent exchange transfusion; therefore, this study cannot determine whether ultra-high-irradiance phototherapy reduces the need for exchange transfusion. This clinical outcome requires evaluation in a substantially larger prospective cohort.

This study evaluated bilirubin reduction across irradiance levels up to 148 μW/cm^2^/nm in neonates with severe jaundice near the exchange transfusion threshold, demonstrating maintenance of the dose-dependent effect during ultra-high-irradiance phototherapy. In addition to irradiance, factors associated with the reduction rate, including birth weight and baseline bilirubin, were identified.

## 5. Limitations

This study has several limitations. First, this was a retrospective, single-center study, and treatment intensity was selected according to clinical judgment; therefore, confounding by indication and residual confounding cannot be excluded. Second, irradiance was estimated from manufacturer specifications and Lambert’s cosine law rather than measured directly at the infant skin surface, and the actual delivered irradiance may therefore have differed from the estimated values. In addition, NeoBlue and Bililux were used during partly different periods of the 8-year study, so device-related findings may also reflect temporal changes in clinical practice. Third, follow-up after discharge was incomplete and nonuniform, which may have introduced selection bias. In addition, ocular effects and other uncommon or long-term adverse events were not systematically assessed; therefore, long-term safety could not be fully evaluated. Fourth, the etiology of hyperbilirubinemia could not be fully classified in all infants, and potentially relevant factors such as feeding volume, hydration status, albumin concentration, and severity of hemolysis were not comprehensively adjusted for. Fifth, total serum bilirubin values within 24 h before the commencement of phototherapy were missing for four infants. These values were replaced by transcutaneous bilirubin levels, which may introduce measurement bias into the findings. Finally, the sample size was not determined before data collection. However, the estimated sample size required to detect a medium effect size (f^2^ = 0.15) with an alpha of 0.05 and 80% power was 55 patients, supporting the adequacy of the current sample size of 92 patients. Nonetheless, the overall sample size and several small treatment subgroups limited the assessment of exchange transfusion, rare adverse events, and device-specific differences, and the single-center setting may limit generalizability to other populations and clinical settings.

## 6. Conclusions

In severe neonatal jaundice near the exchange transfusion threshold, ultra-high-irradiance phototherapy, with estimated irradiance levels up to 148 μW/cm^2^/nm, was associated with a greater bilirubin reduction rate. Prospective multicenter studies with direct irradiance measurement and systematic long-term follow-up are needed to confirm the efficacy and safety of ultra-high-irradiance phototherapy.

## Figures and Tables

**Figure 1:**
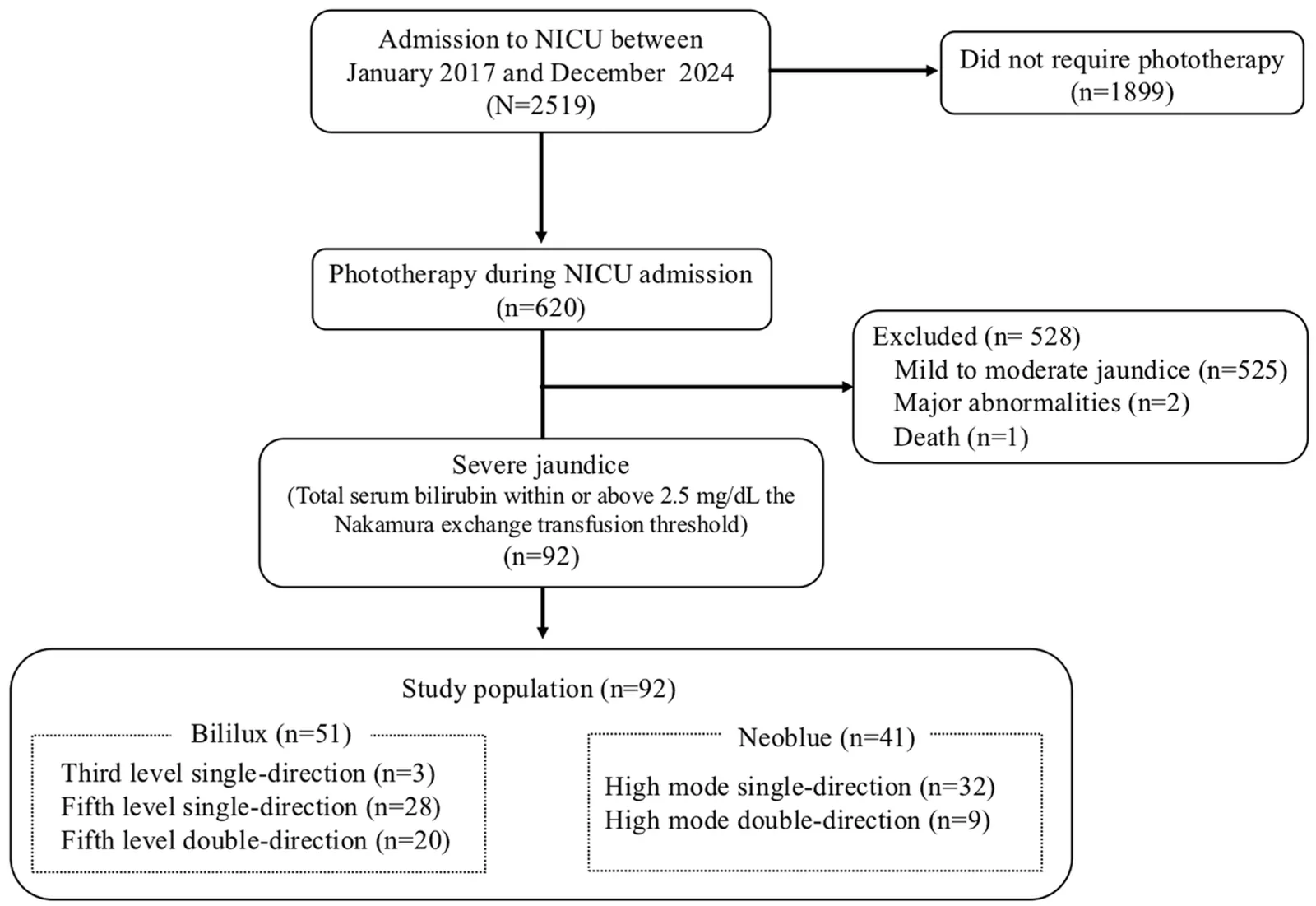
Flow diagram of participant enrollment.

**Figure 2:**
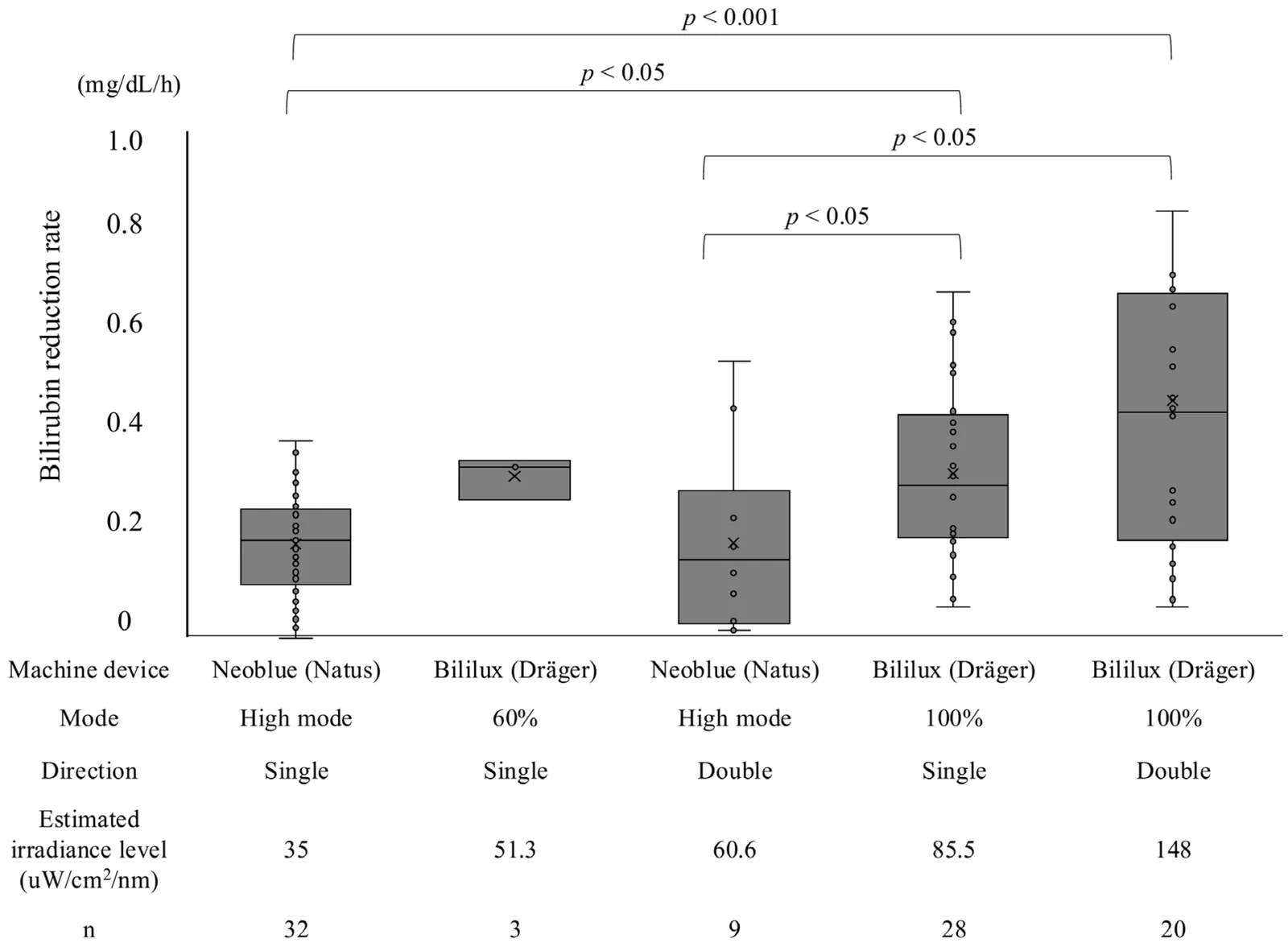
Comparison of the bilirubin reduction rate per device and phototherapy intensity.

**Table 1:** Exchange transfusion thresholds according to the Nakamura criteria.

Birth Weight	<24 h	<48 h	<72 h	<96 h	<120 h
≥2500 g	≥12 mg/dL	≥18 mg/dL	≥20 mg/dL	≥22 mg/dL	≥25 mg/dL
1500–2499 g	≥10 mg/dL	≥15 mg/dL	≥18 mg/dL	≥20 mg/dL	≥20 mg/dL
1000–1500 g	≥10 mg/dL	≥12 mg/dL	≥15 mg/dL	≥15 mg/dL	≥18 mg/dL
<1000 g	≥8 mg/dL	≥10 mg/dL	≥12 mg/dL	≥12 mg/dL	≥15 mg/dL

**Table 2:** Characteristics of the study population.

Variables	Incidence (%)	Mean or Median (IQR)	Standard Deviation
Gestational age, weeks		38.1 (37.0–39.3)	
Birth weight (g)		2704	620
Sex (Male)	54 (59%)		
Mode of delivery			
Caesarean section	16 (17%)		
Apgar score			
1 min		8 (7–8)	
5 min		9 (8–9)	
Umbilical cord blood pH		7.29 (7.27–7.32)	
Feeding			
Breast milk	54 (59%)		
Infusion solution	61 (66%)		
Body weight loss from birth to the commencement of PT (%)		−3.7 (−5.3 to −1.0)	
Hemolytic disease	7 (8%)		
Age at T_−24_ (h)		51.9	36.6
TSB at T_−24_ (mg/dL)		11.7	3.9
Age at T_0_ (h)		74.6	47.2
TSB at T_0_ (mg/dL)		17.7	3.9
Duration of T_−24_ and T_0_ (h)		22.8	18.7
TSB difference between T_−24_ and T_0_ (mg/dL)		6.0	2.6
Bilirubin increase rate (mg/dL/h)		0.36	0.21
Age at T_+24_ (h)		95.1	47.6
TSB at T_+24_ (mg/dL)		12.3	2.6
Duration of T_0_ and T_+24_ (h)		36.9	4.4
TSB difference between T_0_ and T_+24_ (mg/dL)		5.4	3.4
Bilirubin reduction rate (mg/dL/h)		0.24	0.23
Difference between TSB and ET threshold (mg/dL)		−0.72	2.0
PT			
Machine device: Bililux (Dräger)	51 (55%)		
Irradiance level (μW/cm^2^/nm)		77.7	42.4
Exchange transfusion	1 (1%)		
Complications of PT			
Hyperthermia	11 (12%)		
Oliguria	0 (0%)		
Excessive body weight loss	4 (4%)		
Abnormal AABR	1 (1%)		
Cancer	0 (0%)		
Follow-up period (months)		31.5	23.9

Data are presented as numbers (%), means ± standard deviations, or medians (interquartile ranges). Abbreviations: T_−24_, the day before PT; T_0_, the commencement of PT; T_+24_, the day after PT; TSB, total serum bilirubin; PT, phototherapy; ET, exchange transfusion; AABR, automated auditory brainstem response.

**Table 3:** Crude association between the bilirubin reduction rate and clinical variables.

Variables	Regression Coefficient			*p*-Value
	Mean	95% CI		
		Lower	Upper	
Gestation	0.013	−0.002	0.026	0.081
Birth weight (per 100 g)	0.008	0.001	0.015	0.050
Sex (Male)	0.001	−0.071	0.073	0.991
Mode of delivery				
Caesarean section	−0.054	−0.146	0.039	0.253
Apgar score				
1 min	0.011	−0.009	0.031	0.270
5 min	0.019	−0.008	0.047	0.173
Umbilical cord blood pH	0.089	−0.393	0.571	0.715
Feeding				
Breast milk	0.029	−0.043	0.100	0.427
Infusion solution	−0.042	−0.116	0.032	0.265
Body weight loss from birth to the commencement of PT	0.005	−0.003	0.012	0.220
Hemolytic disease	−0.029	−0.162	0.104	0.665
Age at T_0_ (h)	0.002	0.001	0.002	<0.001
TSB at T_0_ (mg/dL)	0.031	0.024	0.037	<0.001
Bilirubin increase rate (mg/dL/h)	0.056	−0.110	0.221	0.504
Age at T_+24_ (h)	0.001	0.001	0.002	<0.001
TSB at T_+24_ (mg/dL)	0.003	−0.010	0.017	0.654
TSB difference between T_0_ and T_+24_ (mg/dL)	0.060	0.052	0.067	<0.001
Difference between TSB and ET threshold (mg/dL)	0.019	0.002	0.036	0.028
PT				
Machine device: Bililux (Dräger)	0.135	0.069	0.200	<0.001
Irradiance level per 100 μW/cm^2^/nm	0.243	0.141	0.345	<0.001

Abbreviations: T_0_, the commencement of PT; T_+24_, the day after PT; CI, confidence interval; TSB, total serum bilirubin; PT, phototherapy; ET, exchange transfusion.

**Table 4:** Multivariable analysis of the bilirubin reduction rate.

Variables	Regression Coefficient			*VIF*	*p*-Value
	Mean	95% CI			
		Lower	Upper		
Birth weight (per 100 g)	−0.006	−0.012	0.000	1.711	0.040
At T_0_ TSB (mg/dL)	0.040	0.029	0.051	1.91	<0.001
Time point of TSB measurement (h)	0.001	0.000	0.002	2.57	0.034
Machine device: Bililux (Dräger)	−0.017	−0.106	0.071	2.63	0.699
Irradiance level per 100 μW/cm^2^/nm	0.220	0.117	0.324	2.64	<0.001

Abbreviations: CI, confidence interval; TSB, total serum bilirubin; PT, phototherapy.

## Data Availability

The datasets generated or analyzed during this study are not publicly available due to institutional and ethical restrictions regarding patient confidentiality but are available from the corresponding author upon reasonable request.

## Abbreviations

The following abbreviations are used in this manuscript:

NICU	Neonatal intensive care unit
CI	Confidence interval
